# The *dpsA* Gene of *Streptomyces coelicolor*: Induction of Expression from a Single Promoter in Response to Environmental Stress or during Development

**DOI:** 10.1371/journal.pone.0025593

**Published:** 2011-09-30

**Authors:** Paul D. Facey, Beatrica Sevcikova, Renata Novakova, Matthew D. Hitchings, Jason C. Crack, Jan Kormanec, Paul J. Dyson, Ricardo Del Sol

**Affiliations:** 1 Institute of Life Science, College of Medicine, Swansea University, Swansea, United Kingdom; 2 Institute of Molecular Biology, Slovak Academy of Sciences, Bratislava, Slovak Republic; 3 Centre for Molecular and Structural Biochemistry, School of Chemistry, University of East Anglia, Norwich, United Kingdom; University of Kent, United Kingdom

## Abstract

The DpsA protein plays a dual role in *Streptomyces coelicolor*, both as part of the stress response and contributing to nucleoid condensation during sporulation. Promoter mapping experiments indicated that *dpsA* is transcribed from a single, *sigB*-like dependent promoter. Expression studies implicate SigH and SigB as the sigma factors responsible for *dpsA* expression while the contribution of other SigB-like factors is indirect by means of controlling *sigH* expression. The promoter is massively induced in response to osmotic stress, in part due to its sensitivity to changes in DNA supercoiling. In addition, we determined that WhiB is required for *dpsA* expression, particularly during development. Gel retardation experiments revealed direct interaction between apoWhiB and the *dpsA* promoter region, providing the first evidence for a direct WhiB target in *S. coelicolor*.

## Introduction

A common mechanism used by bacteria to selectively modulate gene expression in response to stress involves promoter selection by alternative sigma factors. A paradigm of this regulatory strategy is the stress response regulon controlled by the transcription factor Sigma^B^. Initially described in *Bacillus subtilis*, where it controls expression of around 200 genes in response to osmotic, ethanol and temperature stresses, Sigma^B^ orthologs have been shown to perform similar roles in other Gram positives like *Staphylococcus aureus* and *Listeria monocytogenes* [1].

The soil, a complex environmental niche where most *Streptomyces* species thrive, poses serious challenges to the cell's metabolic balance. Sudden modifications of salinity, moisture and temperature are only a few of these challenges, leading to the activation of complex regulatory networks controlling a myriad of genes involved in stress responses and ultimately allowing adaptation to the harsh surroundings. The response to stress in *Streptomyces coelicolor* has been extensively studied and a central role for a Sigma^B^ ortholog has been identified [2]. Furthermore, the *S. coelicolor* genome encodes 9 Sigma^B^-like paralogs, probably an indication of the complex stress-response strategies imposed by its natural environment [3], [4]. In contrast with *B. subtilis*, where various stress conditions induce a single regulon under the control of Sigma^B^, proteomics studies indicate that in *S. coelicolor* different regulons are activated in response to specific stresses. This led to the interpretation that independent control mechanisms could govern individual stress responses. Interestingly, numerous stress-induced proteins are also developmentally controlled, suggesting a dual role for regulatory elements involved in both stress responses and development [5].

The multiple Sigma^B^-like paralogs encoded by *S. coelicolor* support the idea of ‘one Sigma^B^-like paralog per stress type’, but this notion has been consistently challenged as further genetic and gene expression data describing Sigma^B^-like sigma factors has accumulated. The characterisation of *sigH* expression revealed the presence of several promoters induced by heat, osmotic stress and developmental stage [6]. Moreover, *sigH* is also under the control of BldD, which represses its expression during vegetative growth [7]. In addition to *sigH*, several of the *S. coelicolor* Sigma^B^-like sigma factors are also induced by osmotic stress (*sigB*, *L*, *I*, *K* and *M*) while others are mainly involved in morphogenesis (*sigF*, *sigN*). The activation of multiple sigma factors in response to a specific stress suggests the existence of a much more complex and overlapping regulatory network [8]. Experimental evidence resulting from *in vitro* transcription experiments indicates that members of the *S. coelicolor* Sigma^B^ family can recognise similar promoters [4], leading to the assumption that they have overlapping promoter specificities. In contrast to this *in vitro* evidence, most of the SigB-like factors in *S. coelicolor* are apparently quite specific at recognising promoters and are usually autoregulated, as observed when analysing the expression of target genes in the corresponding *sigB*-like mutants. SigH has been shown to direct transcription of one of its own promoters as well as *ssgB*, *gltB* and s*igJ* (SCO1276), and in all cases their expression is dramatically reduced in a *sigH* mutant [9]–[12]. A similar behaviour was observed when analysing the expression of *sigB* and several of its targets identified from bioinformatics and transcriptomics analyses [13], [2], while SigN is autoregulated and controls the *in vivo* expression of the morphogenetic protein NepA [14]. Furthermore, a regulatory cascade of Sigma^B^-like factors has been inferred from microarray experiments. Based on induction timing in response to osmotic stress control hierarchies of *sigI*→*sigB*→*sigM* and *sigK*→*sigH*→*sigL* sequence were proposed [8], although a similar study by Lee and colleagues implicates *sigB* (referred to as *sigJ* by some authors) as a master regulator, acting at the beginning of a putative cascade consisting of *sigB*→*sigL*→*sigM* [2]. The latter is further supported by a genome wide search using a consensus SigB-dependent promoter sequence, which identified several putative SigB targets including SigL [13].

We have recently described the functional role of DpsA; a nucleoid associated protein whose expression is strongly induced by osmotic and heat stresses. DpsA also contributes significantly to nucleoid condensation during reproductive growth in *S. coelicolor*, together with its two paralogs DpsB and DpsC [15]. Our initial expression analyses did not reveal a clear dependence between *dpsA* expression and SigB, despite the fact that DpsA orthologs are part of stress regulons in other bacteria (*E. coli*, *M. smegmatis*) and in *Bacillus subtilis* its expression is part of the general stress response controlled by SigB [16].

Here we describe how *dpsA* is regulated in response to stress and during development. Stress-dependent expression of *dpsA* is dependent on a regulatory cascade involving SigB-like sigma factors in *S. coelicolor*. A single promoter drives *dpsA* expression and is a target for both SigB and SigH. We also identify a role for DNA supercoiling and the WhiB transcription factor in regulation of *dpsA*, indicating how developmental and stress-dependent regulation mediated by these sigma factors can be finessed from a single promoter.

## Results

### *dpsA* is transcribed from a single SigB-like dependent promoter

**Figure 1:**
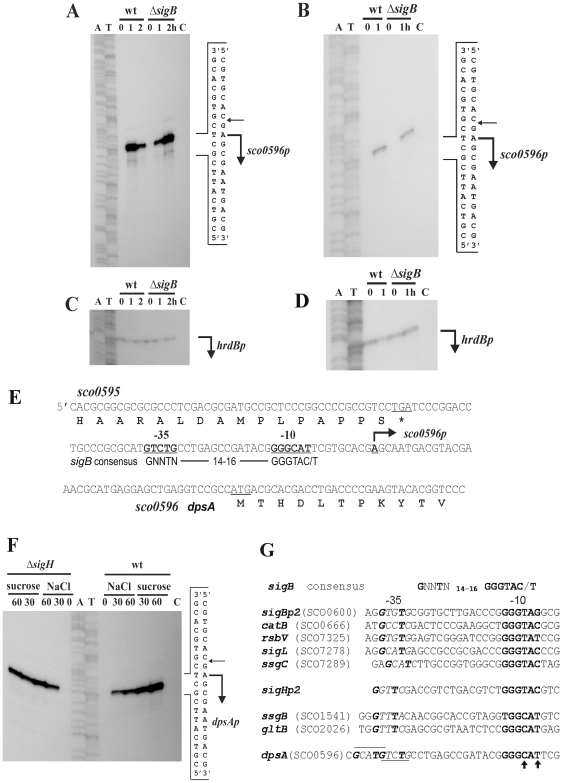
High-resolution S1-nuclease mapping the transcription start point (TSP) of *S. coelicolor dpsA* (*SCO0596*). Bent arrows indicate the positions of RNA-protected fragments. **A**: Total RNA from *S. coelicolor* M145 and its isogenic *sigB* mutant grown for 17 h on a cellophane disc on top of MS agar (lane 0), then transferred to MS containing 250 mM KCl for 1 h (lane 1) and 2 hours (lane 2). **B**: Total RNA from cells grown as above but transferred for 1 h to 42°C (lane 1). **C** and **D**: Control S1-nuclease mapping experiments with the same RNA samples using a DNA probe for the *hrdBp* promoter. E: Nucleotide sequence of *S. coelicolor* M145 *SCO0596* promoter region. The deduced protein product is shown below. The TSP of the *SCO0596* promoter is indicated by the bent arrow. The proposed −10 and −35 boxes of the promoter are in bold characters and underlined. **F**: S1-nuclease protection assay using RNA isolated from *S. coelicolor* M145 and *sigH* mutant (as indicated above the figure), grown for 20 h in liquid minimal NMP+0.5% mannitol medium (lane 0) and osmotic stress induced by addition of NaCl (final concentration 0.5 M) or sucrose (final concentration 1 M) and incubated for 30 min (lane 30) and 60 min (lane 60). In all cases lane C is *E. coli* tRNA, used as negative control **G**: Promoter sequences of consensus *sigB*-like, *sigBp2*, *sigHp2* and several promoters known to be controlled by SigB or SigH respectively. The underlined sequence in *dpsA* promoter indicates putative −35 sequences and arrows indicate modifications from consensus −10 sequence.

Our initial studies using both Quantitative Real-Time PCR (qRT PCR) and immunoblots revealed that *dpsA* expression is strongly up-regulated in response to osmotic up-shift and high temperature [15]. High resolution S1 protection assay experiments performed using total RNA extracted from *S. coelicolor* M145 grown in MS agar and MS agar containing 250 mM KCl confirmed that *dpsA* expression is strongly induced by osmotic stress from a single transcription start point (Figure 1A and C), while transcripts are almost undetectable in the non-stressed sample. Similar experiments using cells grown under heat shock (42°C) also showed induction of expression from the same transcription start point (Figure 1B and D). In both cases total RNA samples isolated from a *sigB* mutant grown under the conditions described above were processed in a similar manner, revealing that transcription still proceeds from the same transcription start point (Figure 1A and B). The only noticeable difference between the parental strain and *sigB* mutant is the apparent delay in *dpsA* induction by 250 mM KCl in the latter. While in M145 the highest induction is observed after 1 hour of osmotic up-shift, in a *sigB* mutant comparable induction levels are only reached after 2 hours, which suggests that in the absence of SigB the observed induction could be mediated by another sigma factor which either binds with less affinity to the *dpsA* promoter or is induced to the required levels after longer exposure to high salt concentration. The putative −10 and −35 sequences were identified and shown to resemble Sigma^B^-dependent consensus promoters (Figure 1E). We also explored *dpsA* expression in a *sigH* mutant, as this SigB-like sigma factor is known to be induced by both osmotic stress and heat [6]. Osmotic stress caused by NaCl and sucrose resulted in an increase of *dpsA* transcription in a *sigH* mutant from the single promoter described above (Figure 1F).

Careful examination of the −10 sequence of the *dpsA* promoter reveals subtle differences when compared to its equivalent in SigB-dependent consensus promoters. The consensus −10 sequence GGGTAC/G changes to GGG**C**A**T** (T→C, C/G→T) in *dpsAp*, similar to −10 sequences from genes known to be transcribed by SigH (*ssgB*, *gltB*). This suggests that *dpsA* could be a direct target for SigH regulation, rather than a member of the SigB regulon (Figure 1G). It is also noticeable that there are two putative −35 sequences in *dpsAp*, depending on a spacer length of 14 or 18 nucleotides between the −10 and −35 sequences.

### Stress-induced control of dpsA expression does not depend on a single SigB-like transcription factor

**Figure 2:**
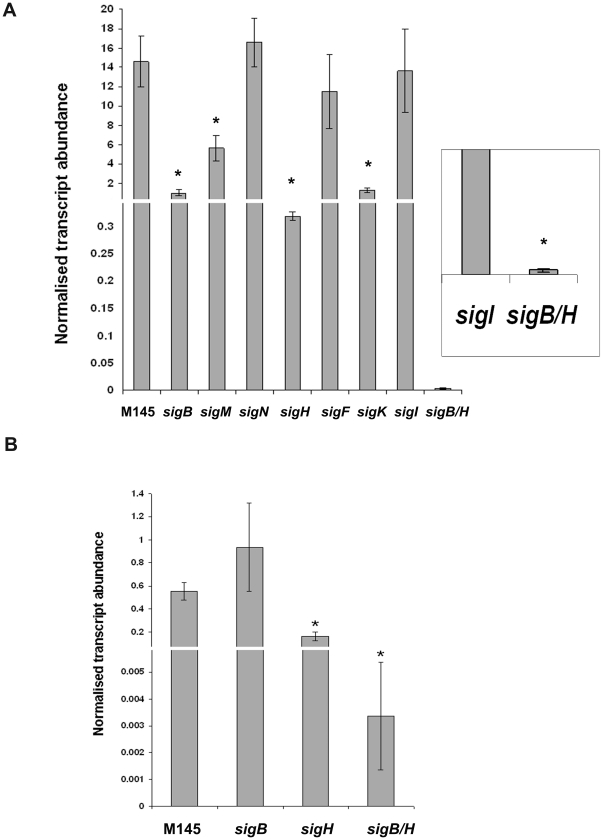
Quantification of *dpsA* transcript abundance in response to osmotic stress. qRT PCR monitoring *dpsA* expression levels after induction by 250 mM KCL in *S. coelicolor* M145 and *sigB*, *sigH*, *sigM*, *sigN*, *sigF*, *sigK*, *sigI* and *sigB*/*H* (inset) mutants (**A**). *dpsA* expression in *S. coelicolor* M145, *sigB*, *sigH* and *sigB/H* mutants after 1 hour of incubations at 42°C (**B**). * indicates significant differences with equivalent *S. coelicolor* M145 sample (One Way Anova, P<0.05). Broken Y axis has been used. Error bars indicate standard deviation.

We used qRT PCR in order to determine precisely how expression levels from the single, *sigB*-like, *dpsA* promoter are affected in *sigB-*like mutants. Total RNA was extracted from *S. coelicolor* M145 (wild type), *sigB*, *sigH*, *sigI*, *sigK*, *sigM*, *sigN*, *sigK*, *sigF* and *sigB*/*H* mutants grown on cellophane discs placed on the surface of MS plates; incubated for 16–18 hours and then transferred to MS plates containing 250 mM KCL. In all cases a set of MS plates was kept as a non-stressed control. Cells were collected after 1 hour of further incubation, RNA extracted and cDNA synthesised. The relative abundance of *dpsA* transcripts was determined by qRT PCR as described [15] using *dpsA* gene specific primers and *hrdB* as internal, normalising control. Basal, non-induced, *dpsA* expression levels are very low and upon osmotic shock *dpsA* transcript abundance dramatically increases in all strains (ranging from 10-fold to 80-fold). The normalised *dpsA* basal expression level for each strain under study was subtracted from the corresponding induced expression levels detected, in order to ensure that only differences in *dpsA* expression resulting from osmotic shock were scrutinised. Wild-type *dpsA* induction levels were observed in *sigI*, *sigN* and *sigF* mutants. Although in both *sigB* and *sigH* mutants *dpsA* was induced by osmotic stress, interestingly transcript abundance never reached the levels attained in the parental strain M145 (Figure 2A) and were particularly low in a *sigH* mutant. Statistical analyses (one way Anova) revealed that the observed differences between the *S. coelicolor* M145 strain and the *sigB*, *sigH*, *sigM*, *sigK* and *sigB/H* mutants were indeed significant. Remarkably, in a *sigB/H* mutant *dpsA* expression remained almost non-induced by osmotic stress, indicating an absolute requirement for both SigB and SigH during *dpsA* osmotic stress up-regulation. No induction was observed in the *sigB*/*H* mutant despite the prolonged incubation under stress (not shown). This result also confirms that both SigB and SigH are responsible for *dpsA* expression and none of the remaining SigB-like elements can replace them functionally.

Since *dpsA* induction levels were significantly affected in a *sigH* mutant background either as a single or double mutant, we further investigated the role of this sigma factor in *dpsA* control. Heat-mediated *dpsA* induction was examined in *S. coelicolor* M145 parental strain and *sigB*, *sigH* and *sigB/H* mutants. *dpsA* expression was up-regulated to similar levels by heat in both M145 and *sigB* mutant strains, although transcript abundance never reached the levels observed during osmotic stress induction. In a *sigH* mutant there is a three-fold reduction on *dpsA* induction level after 1 hour at 42°C when compared to the M145 strain, while in a *sigB*/*H* mutant *dpsA* expression is heavily compromised in both control and heat treated samples, reminiscent of the lack of induction by osmotic stress on this double mutant (Figure 2B). It is noticeable that while SigB is required for full *dpsA* osmotic stress induction, the lack of this sigma factor alone does not affect heat-induced activation of the *dpsA* promoter, which is dependent on a functional SigH.

**Figure 3:**
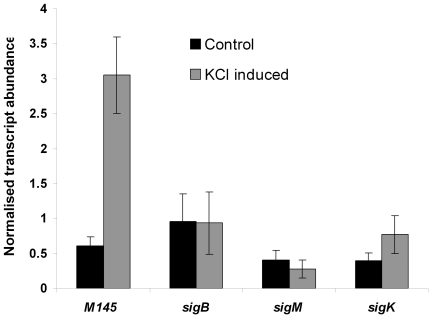
*sigH* osmotic induction depends on SigB-like factors. qRT PCR monitoring *sigH* expression levels after induction by 250 mM KCL for 1 hour in *S. coelicolor* M145, *sigB* ^−^, *sigM* ^−^, and *sigK* ^−^ strains. Error bars indicate standard deviation.

It is evident from the above results that SigH plays a key role in *dpsA* stress mediated induction, although this role may be modulated by SigB or other SigB-like factors. We used qRT PCR to assess the expression of *sigH* after osmotic stress in the parental strain and in different mutant strains deficient in SigB-like sigma factors known to be induced by osmotic stress [8]. A marked *sigH* induction by KCl was observed in the parental M145 strain, while this induction was abolished or reduced in *sigB*, *sigM* and *sigK* mutants (Figure 3), suggesting that these sigma factors influence directly or indirectly *sigH* osmotic induction. Basal *sigH* transcript abundance remained within comparable levels, unaffected by the loss of the SigB-like factors. Combined with the *dpsA* expression studies described earlier (Figure 2A), these results provide an explanation for reduced *dpsA* osmotic induction observed in the *sigM* and *sigK* mutants, due to a reduced induction of *sigH*, which is required for proper *dpsA* activation. The observed reduced *sigH* expression in the *sigB* mutant is likely both direct and indirect, as the latter is able to drive *dpsA* induction in the absence of SigH but is also required for *sigM* expression [2] and putatively for *sigH* expression as suggested by the presence of a SigB-consensus promoter in *sigH* (*sigHp2*, Figure 1G) and our own observations.

This cascade explains the reduced *dpsA* expression in a *sigB* mutant, where reduced *sigH* expression results in diminished *dpsA* activation. As disruption of *sigH* does not completely abolish *dpsA* osmotic induction, an alternative sigma factor functionally replaces SigH. SigB is a plausible candidate, as *dpsA* induction is totally abolished in a *sigB/H* double mutant.

### Inhibition of DNA gyrase affects *dpsA* osmotic stress induction

**Figure 4:**
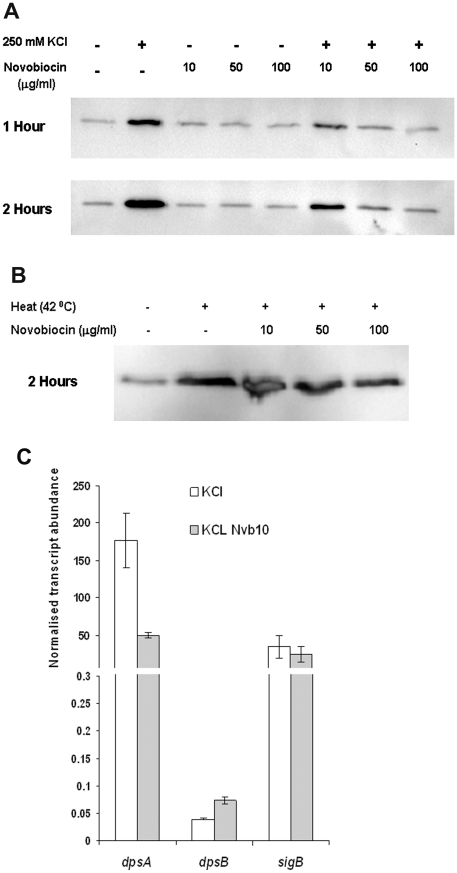
Negative DNA super-coiling contributes to *dpsAp* induction by osmotic stress. **A**: Increasing concentrations of novobiocin abolish DpsAHis induction in the presence of osmotic stress but basal expression levels remain unchanged. **B**: Heat dependent induction of DpsAHis is independent of novobiocin treatment. *S. coelicolor dpsA* ^−^/pDpsA7H was used for both experiments. Time indicates incubation period under stress in the presence or absence of novobiocin at concentrations shown. **C**: qRT PCR showing decrease in salt-induced *dpsA* transcript abundance in response to novobiocin treatment. *dpsB* and *sigB* transcript abundance under the same conditions was also determined. Error bars indicate standard deviation.

Osmotic stress can result in an increase in negative DNA supercoiling, and the change in DNA topology can directly modify transcription of specific genes [17], [18]. Novobiocin, a gyrase B inhibitor, was used to determine if an increase in negative DNA supercoiling resulting from osmotic stress contributed (directly or indirectly) to DpsA up-regulation, monitored using a C-terminal translational fusion to 6 Histidines under the control of *dpsA* native promoter. Western blot experiments revealed that DpsA basal expression levels remained unchanged as a result of novobiocin treatment (Figure 4A), indicating that constitutive expression is insensitive to gyrase B inhibition. In contrast, osmotic stress induction was significantly modified by the antibiotic in a dose dependent manner. At the lowest concentration of 10 µg/ml novobiocin some up-regulation of DpsA was observed, although lower than in the untreated control. At higher concentrations DpsA abundance remained similar to non-induced levels (Figure 4A). This indicated a requirement for active gyrase B and hence an increase in negative DNA supercoiling for induction of *dpsA* expression after osmotic stress. A similar experiment analysing heat-stress induction of DpsA revealed that this up-regulation is independent of DNA negative supercoiling, as novobiocin treatment had no apparent effect even after 2 hour incubation (Figure 4B).

To establish that the observed novobiocin effect was specific to *dpsA* expression and not a consequence of global reorganisation of gene expression due to reduced DNA supercoiling, we grew *S. coelicolor* M145 on MS agar for 16 h and then transferred it to MS agar/250 mM KCL and MS agar/250 mM KCL/10 µg/ml novobiocin. Total RNA was isolated, converted to cDNA and used as template to perform qRT PCR to quantify *dpsA* transcript levels. As negative controls we monitored the expression levels of *dpsB* and *sigB*. The novobiocin treatment caused a reduction in *dpsA* transcript abundance by a third as compared with the untreated sample, while expression levels of *dpsB* and *sigB* remained unaffected, confirming that *dpsA* expression is indeed influenced by topological changes in DNA (Figure 4C). As an additional control, we quantify the expression of 16S rRNA transcript, which remained also unaffected by the novobiocin treatment even at the higher concentrations (not shown).

### Developmental control of *dpsA* expression depends on SigH and SigB and requires WhiB

**Figure 5:**
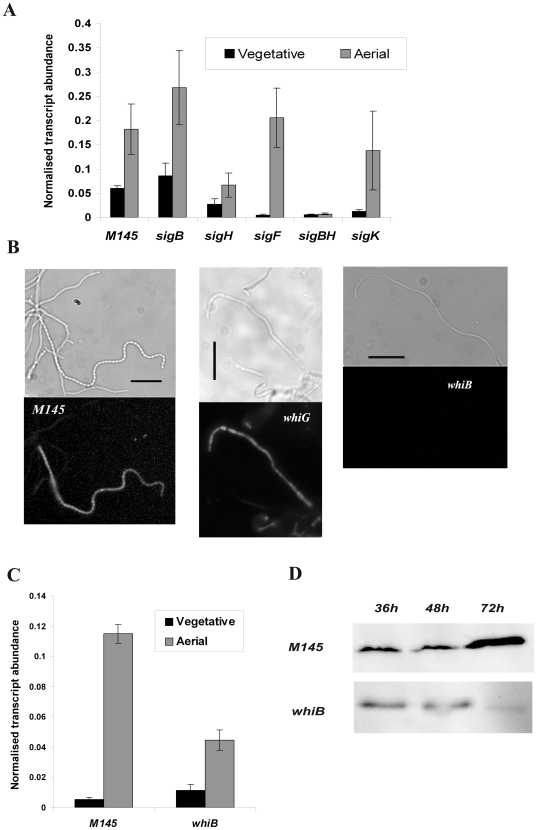
Developmentally controlled expression of *dpsA* in *S. coelicolor* M145, *sigB* ^−^, *sigF* ^−^, *sigK* ^−^ and *sigB*/*H* ^−^ strains assessed by qRT PCR (**A**). Bright field and corresponding fluorescence image showing DpsA_mCh_ expression in aerial hyphae of *S. coelicolor* M145, *whiG* ^−^ and *whiB* ^−^ strains. Bar: 10 µm (**B**). qRT PCR showing *dpsA* transcript abundance in vegetative and aerial hyphae of *S. coelicolor* M145 and *whiB* mutant. Error bars indicate standard deviation. (**C**). Immunoblot comparing DpsAHis abundance in *S. coelicolor* M145 and *whiB* ^−^ strain throughout the developmental life cycle. Similar amounts of total protein were loaded in each lane (**D**).

The *dpsA* gene is developmentally controlled, as its expression is drastically up-regulated during sporulation [15]. SigH is an obvious candidate to modulate such up-regulation as it is known to exert developmental control [10] and is also required for normal aerial development [9]. We performed qRT PCR experiments to monitor and compare *dpsA* expression in vegetative and aerial hyphae from *S. coelicolor* M145 and mutant strains (*sigH*, *sigK* and *sigF*, all of which are known to be involved in aerial development). We also included *sigB* and *sigB/H* mutants. The *sigN* mutant was not included in this study because our previous work [15] showed that *dpsA* expression is not supported in the sub-apical compartment, opposite to what has been described for the SigN target *nepA* [14]. Developmentally controlled expression of *dpsA* in *sigB*, *sigK* and *sigF* mutants remained similar to that of the parental M145 strain, while a reduced expression was detected in *sigH* aerial hyphae. Remarkably, developmental up-regulation of *dpsA* was abolished in a *sigB/H* mutant, reminiscent of the absence of stress induction in this mutant strain and indicating that no other sigma factor can replace SigB or SigH (Figure 5A).

We used a *dpsA*:*mCherry* translational fusion (DpsA_mCh_) under the control of the *dpsA* native promoter to determine *in situ* DpsA expression in aerial hyphae from two early *whi* mutants (*whiG* and *whiB*). An integrative PhiC31-derived plasmid (pDpsA6A) carrying the fusion was conjugated into the mutants under study and the expression of DpsA_mCh_ visualised using fluorescence microscopy as described [15]. Interestingly, while a *whiG* mutant supports normal *dpsA* expression in aerial hyphae, a requirement for a functional WhiB was detected as we could not visualise red fluorescence due to DpsA_mCh_ in this mutant (Figure 5B). This result was corroborated by a qRT PCR experiment using RNA extracted from *whiB* aerial hyphae that revealed reduced *dpsA* expression in this mutant (Figure 5C). Although reduced, *dpsA* mRNA expression levels in aerial hyphae are higher than those observed in vegetative cells. This suggests that additional mechanisms, perhaps involving post-translational DpsA processing, are in place in a *whiB* mutant. We further analysed this WhiB dependence by introducing into the *whiB* mutant an integrative plasmid encoding a *dpsA_His_* translational fusion under the control of the *dpsA* native promoter (pDpsA7A, [15]). Total proteins were isolated from the resulting strain after growth on MS agar at different time points until aerial development was evident. Similar amounts of total protein were assessed by Western blot using an anti-His antibody in order to monitor the abundance of DpsA_His_. A clear reduction of DpsA_His_ levels was observed after the onset of aerial growth (72 hours), confirming our observation of reduced DpsA levels in *whiB* aerial hyphae (Figure 5D).

**Figure 6:**
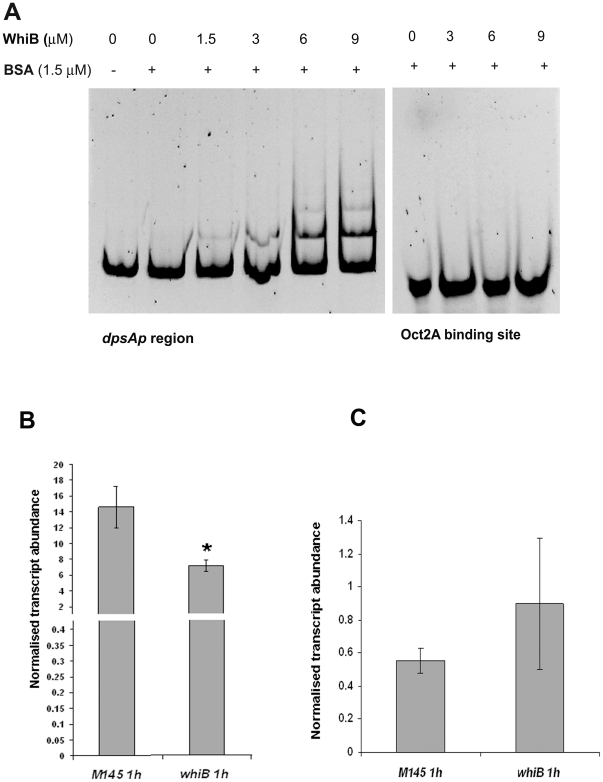
Increasing concentrations of apoWhiB causes electrophoretic shift of *dpsAp* region. A double-stranded oligonucleotide containing the OctA2 binding site was used as negative control (**A**). qRT PCR monitoring *dpsA* expression levels in *S. coelicolor* M145 and *whiB* mutant in response to osmotic stress (**B**) and heat stress (**C**). Significant differences in *dpsA* expression levels (One way Anova, P<0.05) were detected between *S. coelicolor* M145 and *whiB* ^−^ strain after 1 hour of osmotic stress (*). Error bars indicate standard deviation.

Evidence for direct interaction of WhiB with the *dpsA* promoter region was provided by gel retardation experiments. A PCR fragment, encompassing a region from the *dpsA* start codon up to 417 bp upstream DNA sequence, was amplified using primers P1DpsAF1 and P1DpsAR1 and 20 ng were mixed with recombinant apoWhiB at various concentrations (0–9 µM). After 30 minute incubation at room temperature the protein-DNA mix was electrophoresed in a 6% Acrylamide gel, followed by Syto9 staining (Electrophoretic Mobility Shift Assay, Invitrogen). The stained gels were visualised under UV light and an image recorded. As negative control a parallel experiment was carried out using a 39-mer oligonucleotide containing the binding site for Oct2A (Roche). Figure 6A shows the shift in electrophoretic migration of the *dpsA* promoter region caused by apoWhiB, while the negative control remained unaffected.

We also tested *dpsA* KCl-mediated induction in a *whiB* mutant using qRT PCR. The induced levels of *dpsA* transcript in the *whiB* ^−^ strain are lower (∼two fold reduction) than in the parental M145 strain (Figure 6B). This result indicates that the loss of a functional WhiB protein affects *dpsA* osmotic induction, albeit mildly. The fact that a noticeable *dpsA* osmotic induction is still detected suggests that other factors may functionally replace WhiB or that WhiB only has a minor contribution to the *dpsA* up-regulation by osmotic stress. We failed to detect up-regulation of *whiB* transcript in response to osmotic stress, or a dependence on SigB or SigH (not shown). This suggests that the contribution of WhiB to *dpsA* induction during osmotic stress is independent of SigB and SigH control, and constitutes an additional regulatory switch. Similar experiments revealed that WhiB is not required for heat dependent induction of *dpsA* (Figure 6C), confirming earlier observations that indicate the existence of alternative regulatory strategies to activate *dpsA* in response to different stresses.

## Discussion

The osmotic stress response in *Streptomyces coelicolor* is a complex process involving numerous regulatory elements among which SigB-like sigma factors play a central role, together with their cognate anti and anti-anti sigma factors. This network is only superficially understood, as the existence of co-regulation and interaction among its components makes it a challenging puzzle to assemble. Available experimental evidence indicates that SigB may act as a ‘master regulator’ of the osmotic stress response while regulating the expression of many genes, among which there are at least two SigB-like sigma factors, SigL and SigM [2].

Our initial studies characterising *S. coelicolor* Dps paralogs revealed a clear link between *dpsA* expression and the osmotic stress response [15], corroborating previously published data reporting *dpsA* as a target for regulation by SigB [2]. Indeed, promoter mapping experiments confirmed the existence of a single promoter driving *dpsA* expression resembling SigB-like dependent promoters, in particular those transcribed by SigH. Interestingly the *dpsA* promoter is dependent on both SigB and SigH for full induction by high osmolyte concentration, which in turn is abolished in a *sigB*/*H* double mutant. Other SigB-like factors like SigM and SigK are also needed to achieve full *dpsAp* osmotic induction, as its expression is significantly reduced in the corresponding mutants. Analysis of *sigH* expression in response to stress revealed a dependence on various *sigB*-like sigma factors, namely SigB, SigM and SigK. This expression profile supports the proposed cascade governing SigB-like sigma factors in *S. coelicolor*, where SigB acts early on in response to osmotic stress and regulates the expression of its targets among which is *sigM*, which in turns controls *sigH*. The existence of a *sigH* promoter identical to those recognised by SigB strongly supports the existence of a direct regulatory link between SigB and *sigH* expression as well, as shown by our experiments. The up-regulation of *dpsA* expression by heat is mainly dependent on SigH, in contrast with the dual regulation exerted by both SigB and SigH during osmotic stress. This difference can be explained by the presence of a heat inducible promoter of *sigH*, driving expression independently from the salt stress induced *sigHp2* promoter that we propose is controlled by SigB.

**Figure 7:**
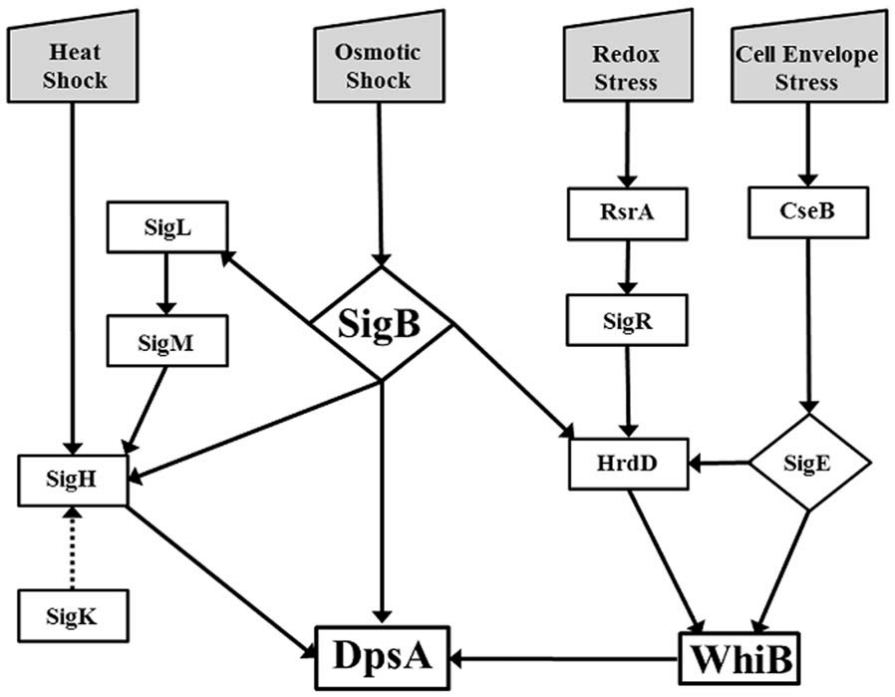
Model depicting the regulatory network controlling *dpsA* expression. Only stress induced elements are shown. Solid lines indicate experimentally verified relationships (direct and indirect) while the dotted line indicates a partially verified one.

Our data suggests that *sigH* is also regulated by SigK, although it is not possible to determine if it is a direct or indirect control. Similarly, we cannot overlook the possibility of SigB-dependent control of *sigK* expression, although the published or available transcriptomics experiments analysing expression in a *sigB* mutant have so far failed to provide evidence for such connection ([2]; Stanford Microarray Database). We explored this idea by monitoring *sigK* expression in *sigB* and *sigH* mutants using qRT PCR, but failed to detect any differences on *sigK* expression levels in response to osmotic stress in those strains (not shown). The lack of *dpsA* induction by high osmolyte in a *sigB/H* double mutant further reinforces our interpretation that SigH and SigB are the main modulators of *dpsA* expression. SigH likely regulates *dpsA* directly, as they are both induced by the same signals (salt stress, heat and development). In contrast to *sigH*, *dpsA* has a single promoter, so the only possible way it can share inducing signals (osmotic and heat stresses) with *sigH* is by being its direct target. SigB is able to replace SigH and drive *dpsA* induction, although less efficiently possibly due to having less affinity for the promoter sequence. A model for this regulatory cascade places SigB as a main modulator of various sigma factors (SigL, SigM, SigH), which results in indirect regulation of *dpsA* expression, but also acting directly on the *dpsA* promoter, although less efficiently. This multilayered regulatory network allows the integration of multiple signals leading to the activation of a specific gene. SigH may act as a node integrating multiple signals and mediating expression of specific genes in response to various stresses (Figure 7).

Various authors have suggested that SigB-like factors have overlapping promoter specificities, in other terms are able to ‘cross talk’. To our knowledge the experimental evidence for the cross talk is based on *in vitro* transcription experiments on the *B. subtilis* promoter *Pctc*, which can be transcribed by both SigB and SigH [4]. Our experimental work confirms that at least two SigB-like factors (SigB and SigH) modulate *dpsA* induction directly, offering the first *in vivo* experimental evidence for this ‘cross talk’.

The need for stimulus-specific responses to an extremely variable and nutrient depleted environment like the soil calls for energy efficient mechanisms of stress response. Individual sigma factors reactive to specific challenges and able to mediate transcription of defined regulons could permit fine-tuning of specific stress responses. On the other hand, diverse stimuli may require the induction of stress response elements able to protect the cell in a variety of ways. DpsA is one such element, able to protect against oxidative stress by preventing free radical formation and shielding DNA from damage, while contributing to DNA condensation during sporulation [15]. Our data supports the idea of *dpsA* been modulated primarily by SigH, but its activation in response to stress can also be mediated by SigB. Rather than relying on gene expression driven from multiple promoters, each recognised by specific sigma factors and induced by specific stimuli; a sigma factor cascade modulates and integrates various environmental signals. This leads to the induction of well defined and specialised regulons, but is flexible enough to converge in a global stress response element like DpsA. The role of sigma factor antagonists within this model should also be considered, and must be included in future experimentation attempting to offer a more complete view of the regulatory network controlling stress response in *Streptomyces*.

Although *dpsA* is induced by both osmotic up-shift and heat in a SigH dependent manner, the resulting expression levels are very different for each stimulus, namely much higher for salt induced expression. We found evidence for a mechanism that contributes to the difference in induction levels, providing an additional layer of gene expression control. Differences between osmotic and heat stress induction of *dpsA* are in part the result of changes in DNA topology, in particular an increase in negative supercoiling dependent on the activity of gyrase B. An increase in negative supercoiling as a result of osmotic stress is well documented and topoisomerase gene promoters are sensitive to changes in DNA topology. The presence of two potential −35 sequences in *dpsAp* (separated from the −10 sequence by 14 and 18 nucleotides respectively) may explain such topology sensitivity. An increase in negative supercoiling in response to osmotic stress may bring the closer −35 sequence out of phase from the −10, affecting recognition by the corresponding sigma factor. The existence of a second −35 sequence further apart would compensate for the change in promoter topology, as it will ensure that a suitable −35 is always in phase with the −10 sequence and will ensure a sustained expression of *dpsA*. Exploring the relative contribution of each −35 sequence to *dpsAp* activity extends beyond the scopes of this paper, but surely constitutes an exciting proposition. However, the regulation of *dpsA* expression after heat shock is independent of this DNA topology-dependent mechanism, implicating at least two parallel stress-dependent regulatory systems influencing expression of this gene.

Developmental control of *dpsA* expression depends primarily on SigH and SigB. An important finding is the dependence of *dpsA* on WhiB for developmentally controlled expression, the first reported target for this transcription factor in *S. coelicolor*. *dpsA* expression in aerial hyphae was heavily compromised in a *whiB* mutant, and we confirmed *in vitro* binding of apoWhiB to the *dpsA* promoter region. Osmotic induction of *dpsA* is reduced in a *whiB* mutant, expanding the role of this transcription factor beyond a mere development-related switch and into a modulator of gene expression during stress. There are several *whiB* paralogs encoded by *S. coelicolor* genome, and they may also contribute to *dpsA* control during osmotic stress, similar to the combined action of SigH and SigB. We have performed searches on publicly available microarray data (Stanford Microarray Database) and found two *wbl* (*whiB* like) genes whose expression is induced by osmotic stress (SCO5190 and SCO7306) and therefore likely subjects for future studies assessing their potential *dpsA* regulatory role.

These observations fit with published data investigating *in vitro* expression of genes regulated by SigB-like sigma factors. The developmentally controlled gene *nepA* is not transcribed by SigN *in vitro* despite the uncontroversial evidence for *nepAp* dependence on SigN *in vivo* [14]. The *whiEVII* promoter sequence also fails to be transcribed by SigF *in vitro* [19], and in both cases the existence of an additional transcriptional activator has been suggested. We propose that WhiB or a WhiB-like paralog is (are) responsible, together with the corresponding SigB-like factor, for the up-regulation of the above genes in aerial hyphae, just as we observed for *dpsA*. Similarly, the existence of *whiB*-like (*wbl*) genes induced by osmotic stress suggests that they may play a similar role during osmotic stress. Moreover, additional stress factors, such as redox stress or cell envelope stress, are likely to occur concomitantly during conditions of osmotic stress (see Figure 7). In this respect we note that *whiB* can be expressed from one of two promoters [20], [21] and is developmentally regulated by BldD [22]. The first promoter requires HrdD which may be involved in coordinating “cross talk” from osmotic (via SigB), redox (via SigR) and cell envelope (via SigE) stress sensing systems [21], [23], [2]. The second requires SigE, part of a multicomponent system directly involved in monitoring changes in the integrity of the cell envelope [24]. Thus WhiB, like other WhiB-like proteins, presumably senses stress induced changes in the intracellular redox status of the cell via an [FeS] cluster, leading to an enhanced DNA binding affinity [25]–[28]. This would allow fine tuning of *dspA* gene expression as a result of the dual action of the SigB (or SigB-like) and WhiB (or WhiB-like) proteins in response to varying degrees of osmotic stress. We intend to continue exploring the putative connection between SigB-like and WhiB-like factors, particularly the role played by the former in the expression of the latter, in order to identify dependence on each other while controlling their putative gene targets in response to stress and developmental stage.

In summary, we have dissected the *dpsA* expression control mechanism and shown that two sigma factors (SigB and SigH) are able to drive *dpsA* expression in response to stress and during developmental differentiation, and also how expression levels can be modulated by additional transcription factor(s) (WhiB) and DNA topology status. The results described here revealed how a single promoter can be the subject of multiple regulatory factors in response to a variety of stress signals, leading to finely tuned levels of gene expression.

## Methods

### Bacterial strains and media

*Streptomyces coelicolor* A3 (2) and *E. coli* strains are listed and described in Table 1. All cloning procedures were performed in *E. coli* JM109, while *E. coli* ET12567/pUZ8002 was used for intergeneric conjugative transfer of plasmid DNA into *Streptomyces* strains [29]. Gene replacement experiments were performed in BW25113 (pIJ790) strain as described [30]. Culturing of *E. coli* strains was as recommended [31]. *S. coelicolor* strains were grown at 30°C on the surface of MS (mannitol soya flour) agar and/or on cellophane discs [29]. For osmotic up-shock, MS agar was supplemented with 250 mM KCl. *Streptomyces* mutant strains were obtained using Tn*5062*-mutagenised cosmids as described ([32]; Table 1), the double *sigB/sigH* mutant was created by disruption of *sigB* using an apramycin resistant Tn*5062* mutagenised cosmid in an existing thiostrepton resistant *sigH* mutant [9]. The identity of all mutants was confirmed by Southern blot [31].

**Table 1:** Bacterial strains, plasmids and cosmids.

Strain or plasmid	Description	Transposon insertion^a^ (genome position), Genbank accession	Reference/Source
**Strains**			
*S. coelicolor A3(2)* M145	Prototrophic SCP-1 SCP-2 Pgl+		[29]
*DSCO0600, sigB^−^*	M145 *sigB^−^::*Tn*5062 (apra)*	SC5G5.1.C05 (639940)	[35]
*K101*, *sigF^−^*	M145 *sigF^−^::apra*		[14]
*sigH^−^*	M145 *sigH^−^::thio*		[9]
*DSC03068*, *sigI^−^*	M145 *sigI^−^::*Tn*5062 (apra)*	7F11.01.F06 (3361076)	This study
*DSCO7314*, *sigM^−^*	M145 *sigM^−^::* Tn*5062 (apra)*	SC5F8.2.C11 (8120634)	This study
*sigN^−^* (*K100*)	M145 *sigN^−^::apra*		[14]
*sigK^−^*	M145 *sigK^−^::kan*		[36]
*DSCO0600/DSCO5243*, *sigB/H*	M145 *sigB^−^::apra*, *sigH::thio*		This study
*J2402*, *whiB^−^*	M145 *whiB^−^::hyg*		[37]
*J2400*, *whiG^−^*	M145 *whiG^−^::hyg*		[37]
*JM109*	F′ *traD36 proA^+^B^+^ lacIq* Δ*(lacZ)M15/*Δ*(lac-proAB) glnV44 e14- gyrA96 recA1* *relA1endA1 thi hsdR17*		[38]
*ET12567 (pUZ8002)*	*dam13*::Tn*9 dcm6 hsdM hsdR recF143* 16z*jj201* ::Tn*10 galK2 galT22 ara14 lacY1* *xyl5 leuB6 thi1 tonA31 rpsL136 hisG4* *tsx78 mtli glnV44*, containing the non-transmissible*oriT* mobilizing plasmid,pUZ8002		[39]
*BW25113 (pIJ790*	K12 derivative: delta*araBAD*, delta*rhaBAD* containing lambdaRED recombination plasmid pIJ790		[30]
**Plasmids**			
pQM5062	pMOD+Tn5062, Ampicillin^R^ and Aramycin^R^	AJ566337.1	[32]
pDpsA6A	*dpsA*::*mCherry*, ApramycinR		This study
pDpsA6H	*dpsA::mCherry*, HygromycinR		[15]
pDpsA7	*dpsA::His6*, ApramycinR		[15]
pDpsA7H	*dpsA::His6*, HygromycinR		[15]

a Access http://strepdb.streptomyces.org.uk/ for information about transposon insertion details.

### DNA manipulation and plasmid construction

All plasmids are listed in Table 1. DNA manipulation and cloning were carried out following standard protocols [31] using *E. coli* JM109 as a host. Plasmids were verified by restriction analysis and sequencing, and introduced in *Streptomyces* strains by intergeneric conjugation.

An apramycin resistant version of plasmid pDpsA6H [15] was created by replacing the hygromycin-resistance marker using the PCR targeted system [30] with the apramycin gene from plasmid pQM5062 digested HindIII. The resultant plasmid, pDpsA6A, was used for conjugal transfer into hygromycin resistant mutant strains. Plasmid pIJ6999 was used for recombinant expression of WhiB (C. den Hengst, JIC, personal communication). Briefly the *whiB* coding sequence was amplified by PCR using primers WB1 and WB2 (Table 2), which contain NdeI and BamHI recognition sequences respectively. The PCR product was cloned into pET15b, resulting in pIJ6999. PCR amplifications were performed using the high fidelity polymerase *Pfu* (Promega), following the manufacturer's recommendations.

**Table 2:** Oligonucleotides.

Name (target gene)	Sequence (5′ to 3′ direction)
P1DpsAF1	TAGATATCCCATGCTCGGTGAGACCGACG
P1DpsAR1	TACATATGGGACCTCAGCTCCTCATGCG
WB1	GCGCATATGACCGAGCTGGTGCAGC
WB2	GTTGGATCCGCCGCGTGGGGCGGC
hrdBFor	CCTCCGCCTGGTGGTCTC
hrdBRev	CTTGTAGCCCTTGGTGTAGTC
0596RTF1 (*dpsA*)	AGCGGAAGTGGGACGACTAC
0596RTR1(*dpsA*)	TCAGAAGGTCCTCGGTGGC
whiBRTF1	ACCCCGAGTCCTTCTTCC
whiBRTR1	ATTCGGAGCGGACCTCAC
sigHQRT2F	CCCTGGACGACCTGACC
sigHQRT2R	GGAAGTGCCGCTTGATCTC

### High-resolution S1-nuclease protection assay

Promoter mapping experiments were performed as described [9]. The probe used to analyze *dpsA* promoter region was amplified by PCR using a 5′-^32^P-labeled reverse primer SCO0596R (located in the *SCO0596* coding region 100 bp downstream the start codon) and the unlabeled forward primer SCO0569F, binding upstream of SCO0595 gene (ca. 70 bp upstream of *SCO0595* start codon). The single end-labelled DNA fragment was hybridized with 40 µg total RNA, and treated with 100 U of S1-nuclease. The RNA-protected DNA fragments were analyzed on DNA sequencing gels together with G+A (lane A) and T+C (lane T) sequencing ladders derived from the end-labelled fragments [33].

### Purification of apo-WhiB

Soluble apo-WhiB was over produced from plasmid pIJ6999 as a (His)_6_-tagged protein in aerobic *E. coli* cultures (BL21 lambdaDE3 Star, Novagen) using Luria-Bertani (LB) medium supplemented with 100 mg/L ampicillin. Cultures were grown according to Rybniker [28] and induced with 0.5 mM IPTG. Briefly, cell pellets were resuspended in binding buffer (50 mM NaH_2_PO_4_, 200 mM NaCl, 10% (v/v) Glycerol, pH 7.5), treated with 30 mM Imidazole, lysozyme (0.5 mg/ml), DNaseI (0.125 mg/ml), 1.2 mM PMSF, disrupted by sonication and centrigufed at 40,000×g for 45 min at 2°C. Apo-WhiB was isolated under aerobic conditions, via Ni^2+^-NTA affinity chromatography (HisTrap FF Crude, GE-Healthcare) [28]. Bound proteins were eluted (1 ml/min) using a 6 ml linear gradient from 0 to 100% (v/v) elution buffer (50 mM NaH_2_PO_4_, 200 mM NaCl, 500 mM Imidazole, 10% Glycerol, pH 7.5). Fractions (1 ml) containing apo-WhiB were pooled, diluted 10 fold with binding buffer and concentrated using a 1 ml Ni^2+^-NTA column [34]. before being exchanged (PD10, GE-Healthcare) into 50 mM Tris, 100 mM, NaCl 10% (v/v) Glycerol, pH 7.5. Apo-WhiB was stored at −20°C until needed. As isolated, aerobically prepared apo-WhiB was devoid of an iron sulfur cluster (not shown), as previously observed for WhiB2 [28].

### Other Protein methods

Total protein was used for immunodetection of proteins. Cellophane disc cultures were set up as described previously [15] and incubated overnight (∼16 h). To provide osmotic up-shock, overnight cellophane cultures were transferred to MS agar/250 mM KCl and incubated for the specified time. MS agar plates were used as controls. After incubation, mycelia were scraped from the cellophane discs and suspended in Sonication Buffer [50 mM Tris-HCl, pH 8, 200 mM NaCl, 15 mM EDTA, Complete protease inhibitor cocktail (Roche Diagnostics)]. Cells were disrupted by several burst of sonication on ice (20 s at 30% amplitude). Cell-free extracts were obtained by centrifugation (13 000 r.p.m. for 5 min) and recovery of the supernatant. Total protein concentration was determined using the Bradford method (Bio-Rad). SDS-PAGE was performed as previously described [31], loading 10 µg of total protein per lane in 15% SDS PAGE gels. Proteins were transferred to a polyvinylidene difluoride (PVDF) membrane (Hybond-P, Amersham) using a semi-dry electrophoretic transfer cell (Trans-Blot SD, Bio-Rad). Immunological detection was performed using an ECL Advance Western blotting detection kit (Amersham Pharmacia Biotech). His-tagged proteins were detected with a Penta- His peroxidase conjugate (QIAGEN).

### RNA isolation and qRT PCR

Total RNA isolation, reverse transcription and qRT PCR procedures were performed as previously described [15]. Briefly, sterile cellophane cultures were set up as described above. After the required incubation, cells were collected and total RNA isolated with a Qiagen RNeasy mini kit as per the manufacturers' recommendations. cDNAs were obtained from 1 µg of total RNA using a RETROscript reverse transcription kit (Ambion); using the manufacturers recommendations with random decamers in a reaction volume of 20 µl. cDNAs were diluted 1/15 in nuclease free water (Ambion). RT-QPCR was carried out on 5 µl of diluted cDNA with an iCycler iQ real-time PCR detection system (Bio-Rad) using SYBR-Green Supermix 2X containing Thermo-Start DNA Polymerase (ABgene). Gene specific primers used for Quantitative PCR were designed using Beacon Design (Premier Biosoft, USA) and shown in Table 2. The specificity of the reaction was assessed using melt curve analysis. Transcript abundance was determined using the standard curve method against serial dilutions of *S. coelicolor* genomic DNA. *S. coelicolor hrdB* was used as internal control to normalise samples.

### qRT PCR data analysis

Normalised starting quantities were initially tested for normality using the Kolmogorov Smirnov test. Significant differences between transcript abundance among strains were tested using a one-way ANOVA. Dunnett's T3 Test was used post-hoc (equal variances not assumed) to highlight stains that differed most significantly from each other. All statistical analysis procedures were performed in SPSS version 16 for Windows.

### Microscopy

Localisation of *dpsA* expression in mycelia and aerial hyphae was determined using C-termius fusions to mCherry protein and imaged as described [15]. Briefly, spores or mycelia of *S. coelicolor* were inoculated into the acute angle between glass coverslips inserted obliquely into an agar plate and the surface of the medium. Coverslips were removed from the agar and placed onto microscope slides with a drop of 20% glycerol. Preparations were sealed with clear nail varnish and images obtained using a Nikon Eclipse E600 epifluorescence microscope fitted with a Coolsnap microscope camera (RS Photometrics., Tucson, AZ).

### Electrophoretic Mobility Shift Assays

EMSA was performed using the fluorescence based Electrophoretic Mobility-Shift Assay (EMSA) Kit (Invitrogen) according to the manufacturer's instructions. In brief, binding reactions were prepared using recombinant apoWhiB mixed with *dpsA* promoter region amplified by PCR. Reactions were incubated at room temperature for 30 min. BSA was included as a non-specific competitor. Binding reactions were mixed with 1X EMSA gel-loading solution and electrophoresed for 2 hours in 6%, pre-run (90 V 30 min) polyacrylamide gels in 0.5× Tris-Borate/EDTA running buffer. Gels were post-stained for 30 min at room temperature in the dark in 1× TBE containing 1X SYBR® Green EMSA staining solution.
