# Correction: The *dpsA* Gene of *Streptomyces coelicolor*: Induction of Expression from a Single Promoter in Response to Environmental Stress or during Development

**DOI:** 10.1371/annotation/8fedd9bb-d84e-4d7b-bc71-3321e652198d

**Published:** 2011-11-30

**Authors:** Paul D. Facey, Beatrica Sevcikova, Renata Novakova, Matthew D. Hitchings, Jason C. Crack, Jan Kormanec, Paul J. Dyson, Ricardo Del Sol

There was an error in the financial disclosure. The correct financial disclosure statement is: "This work was supported by grant 2/0104/09 from Scientific Grant Agency of the Ministry of Education of the Slovak Republic and the Slovak Academy of Sciences. MDH is supported by a Biotechnology and Biological Sciences Research Council Studentship. JCC was supported by Biotechnology and Biological Sciences Research Council grant BB/G019347/1 to M.J.Butner (John Innes Centre) and N.Le Brun (University of East Anglia). The funders had no role in study design, data collection and analysis, decision to publish, or preparation of the manuscript." 

